# Modulation of Tropane Alkaloids’ Biosynthesis and Gene Expression by Methyl Jasmonate in *Datura stramonium* L.: A Comparative Analysis of Scopolamine, Atropine, and Hyoscyamine Accumulation

**DOI:** 10.3390/life14050618

**Published:** 2024-05-10

**Authors:** Arash Rasi, Manijeh Sabokdast, Mohammad Reza Naghavi, Parisa Jariani, Beáta Dedičová

**Affiliations:** 1 Department of Agriculture and Plant Breeding, Faculty of Agriculture and Natural Resources, University of Tehran, P.O. Box 4111, Karaj 31587-11167, Iran; arashrasi@ut.ac.ir (A.R.); mnaghavi@ut.ac.ir (M.R.N.); parisa.jariani@ut.ac.ir (P.J.); 2 Department of Plant Breeding, Swedish University of Agricultural Sciences (SLU) Alnarp, Sundsvägen 10, P.O. Box 190, SE-234 22 Lomma, Sweden

**Keywords:** alkaloid biosynthesis, *Datura stramonium* L., gene expression, leaves and roots

## Abstract

Scopolamine and atropine are two medicinal alkaloids derived from *Datura stramonium* L. with anticholinergic properties. This study explored how methyl jasmonate (MJ), a plant growth regulator, affects the biosynthesis and accumulation of these alkaloids in different plant tissues. The expression levels of putrescine N-methyltransferase (*PMT*), tropinone reductase I (*TR1*), and hyoscyamine 6β-hydroxylase (*h6h*), three critical enzymes in the biosynthetic pathway, were also analyzed. The results indicated that MJ at 150 µM increased the production of scopolamine and atropine in both leaves and roots, while MJ at 300 µM had an adverse effect. Furthermore, MJ enhanced the expression of *PMT*, *TR1,* and *h6h* genes in the roots, the primary site of alkaloid synthesis, but not in the leaves, the primary site of alkaloid storage. These results imply that MJ can be applied to regulate the biosynthesis and accumulation of scopolamine and atropine in *D. stramonium*, thereby improving their production efficiency.

## 1. Introduction

*D. stramonium*, commonly known as Jimson Weed or Devil’s Snare, is a medicinal plant belonging to the Solanaceae family (Cornelius et al., n.d.; Ryan Supervisor & József Kutszegi, 2022) [1,2]. It has various therapeutic uses, such as alleviating asthma, motion sickness, and Parkinson’s disease (Akbar, 2020; Thapa et al., 2022) [3,4]. The pharmacological effects of *D. stramonium* are mainly due to its alkaloids, scopolamine, and atropine, which act as anticholinergic agents by blocking the muscarinic receptors in the nervous system [5,6,7]. These alkaloids are mainly produced in the roots and then distributed to the aerial parts, such as leaves and flowers (Cinelli & Jones, 2021) [8]. Tropane alkaloids (TAs), including scopolamine and atropine, are critical secondary metabolites with a broad spectrum of therapeutic and pharmacological uses (Dey et al., 2020; Grynkiewicz & Gadzikowska, 2008; Jirschitzka et al., 2013) [9,10,11]. Scopolamine is structurally unique due to an epoxide bridge within its tropane ring, distinguishing it from atropine (Huang et al., 2021; Schlesinger, 2016) [12,13]. Atropine itself is a racemic mixture, comprising the enantiomers (−)-hyoscyamine and (+)-hyoscyamine; however, it is the (−)-hyoscyamine isomer, also known as hyoscyamine, which exhibits pharmacological activity (Kohnen-Johannsen & Kayser, 2019) [14].

These bioactive molecules are primarily produced by plants in the *Solanaceae* family, notably within the genera *Datura*, *Hyoscyamus*, and *Duboisia* (Gutiérrez-Grijalva et al., 2020; Palazón et al., n.d.) [15,16]. While these alkaloids are highly valued for their medicinal properties, their biosynthetic pathways are not fully understood, and their chemical synthesis presents significant challenges. As a result, there is a growing demand for these compounds, sourced directly from natural plant producers.

In the context of tropane alkaloids such as hyoscyamine and atropine, they are celebrated for their mydriatic and spasmolytic properties (Yamada & Tabata, 1997) [17]. The Solanaceae family chiefly synthesizes these substances, with the genera Atropa, Hyoscyamus, and Datura being prominent producers. The biosynthesis of TAs is intricate, involving a cascade of genes, such as *TR1*, *TR2*, *HYOS*, and *SC*, each integral to the biosynthetic pathway.

The tropinone reductase I (*TR1*) and tropinone reductase II (*TR2*) genes encode enzymes, which catalyze the stereospecific reduction in tropinone to produce tropine and pseudo-tropine, respectively (Ullrich et al., 2016) [18]. These enzymes are crucial for the bifurcation of TA biosynthesis, forming distinct alkaloid end products. Recent studies have highlighted the evolution of these genes within the Solanaceae family, suggesting an intricate regulation of TA production.

The *HYOS* gene is associated with the biosynthesis of hyoscyamine, one of the primary TAs with significant medicinal importance. This gene contributes to the early steps of TA biosynthesis, linking primary metabolism to the specialized pathway of TA production.

The SC gene is implicated in the biosynthetic pathway leading to scopolamine, another pharmacologically important TA (Kohnen-Johannsen, 2019; Yang & Stöckigt, 2010) [19,20]. Scopolamine is derived from hyoscyamine through hydroxylation, a reaction facilitated by enzymes encoded by the SC gene.

These genes’ differential expression and regulation contribute to the variability in TA production among different species within the Solanaceae family. By understanding the function and interaction of these genes, we can gain insights into the genetic basis of TA biosynthesis and its evolutionary adaptation across various plant species.

**Figure 1:**
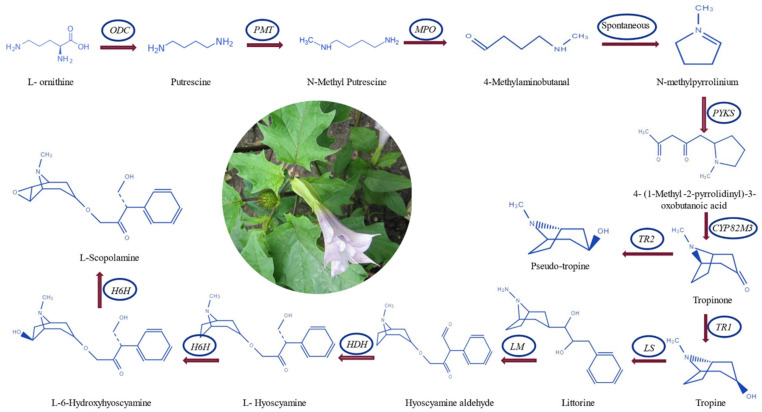
Scopolamine and hyoscyamine biosynthetic pathway in *D. stramonium* [26,27].

The tropane alkaloid biosynthetic pathway is a complex biochemical cascade regulated by key enzymes, including *PMT* and *h6h*, as depicted in Figure 1. *PMT* is responsible for the N-methylation of putrescine, derived from phenylalanine or ornithine metabolism, yielding N-methyl putrescine. Subsequently, *h6h* catalyzes the conversion of atropine to scopolamine through hydroxylation and epoxidation processes. The expression and enzymatic activity are modulated by many factors, encompassing plant tissue specificity, developmental stages, and environmental stimuli. Notably, the biosynthesis and subsequent accumulation of scopolamine and atropine are influenced by environmental conditions and genetic factors, such as light exposure, temperature fluctuations, and the presence of elicitors, as evidenced by studies conducted in Refs (Satish et al., 2020; Ullrich et al., 2016) [18,21].

Among the various elicitors investigated, methyl jasmonate (MJ), a plant hormone integral to physiological regulation and stress response mechanisms, has garnered significant attention. Research in Refs (Ho et al., 2020; Jeyasri et al., 2023; Yu et al., 2018) [22,23,24] has elucidated MJ’s role in modulating the expression of pivotal genes within the tropane alkaloid biosynthetic pathway, including *h6h* and *PMT*. Moradi et al. (Moradi et al., 2020) [25] further demonstrated that MJ’s application can lead to alterations in gene expression, thereby impacting the production of these pharmacologically important alkaloids.

Recent studies have elucidated the multifaceted role of JA as a key regulator of plant growth, development, and defense mechanisms. JA and its derivatives, collectively known as jasmonates, are involved in various physiological processes, including leaf senescence, tuber formation, photosynthesis, reproduction, and seed germination (Sood, 2023; Wasternack & Song, 2017) [28,29]. Furthermore, JA plays a pivotal role in plant responses to biotic and abiotic stress factors, mediating against necrotrophic fungi and alleviating stressors such as drought, salinity, and heavy metal exposure (Macioszek et al., 2023; Pedranzani & Vigliocco, 2017) [30,31]. Regarding secondary metabolite production, JA and MJ have been recognized as significant elicitors in the biosynthesis of tropane alkaloids. Elicitation with these compounds has been shown to alter gene expression levels, particularly those associated with the biosynthetic pathways of hyoscyamine and scopolamine (Nandy et al., 2021; Pourhabibian et al., 2024) [32,33]. For instance, the *h6h* gene, which is involved in tropane alkaloid production, exhibits increased expression levels in response to JA elicitation, correlating with the accumulation of these alkaloids in plant tissues. These insights into the regulatory role of JA and MJ in general physiological processes and specific secondary metabolite pathways provide a solid foundation for understanding their impact on plant biology.

This study aimed to investigate the effects of MJ, at 0, 150, and 300 µM, on the production of scopolamine and atropine in the leaves and roots of *D. stramonium* and to examine the expression levels of *h6h* and *PMT* genes in the roots. The hypothesis was that MJ would increase the production of scopolamine and atropine in both plant organs and that this would be correlated with the upregulation of *h6h* and *PMT* genes in the roots.

It was also expected that the optimal concentration of MJ for eliciting the highest alkaloid yield would differ for each plant organ. The results of this study could provide valuable insights for improving the efficiency and quality of scopolamine and atropine production in *D. stramonium*.

## 2. Results

### 2.1. HPLC Results

**Figure 2:**
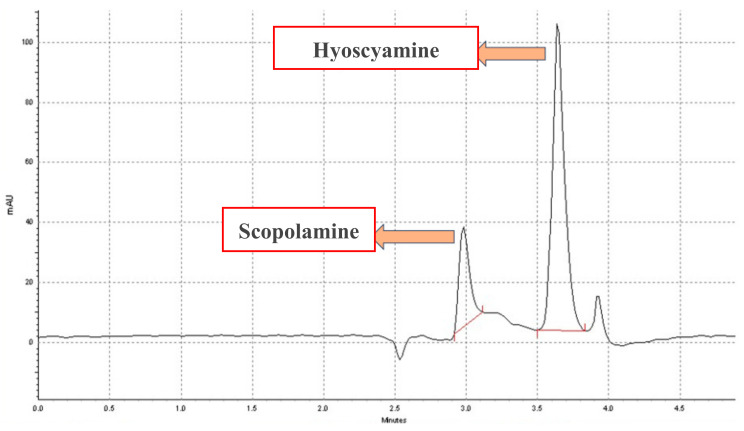
HPLC chromatograms of scopolamine and hyoscyamine in leaf and root tissues of *D. stramonium* plant after 24 h of MJ treatment.

The levels of scopolamine and hyoscyamine, two tropane alkaloids in *D. stramonium*, were measured using HPLC after exposing the plant samples to 0, 150, or 300 µM MJ for 24 h. The alkaloid contents in the leaf and root tissues were quantified separately. The results showed that MJ altered the scopolamine and hyoscyamine content in different tissues of the *D. stramonium* plant after 24 h of treatment (Table 1 and Figure 2). MJ had tissue-specific and concentration-dependent effects on the alkaloid levels in *D. stramonium*. Scopolamine levels in the leaf tissue did not change significantly after treatment with 150 µM or 300 µM MJ, decreasing by 9% and 8%, respectively. However, scopolamine levels in the root tissue increased significantly by 28% and 19% after treatment with 150 µM and 300 µM MJ, respectively, compared to the control. This suggests that MJ stimulated the production and accumulation of scopolamine in the root, which supports the hypothesis that scopolamine is mainly synthesized in the lower parts of the plant. Hyoscyamine levels showed a different pattern.

**Figure 3:**
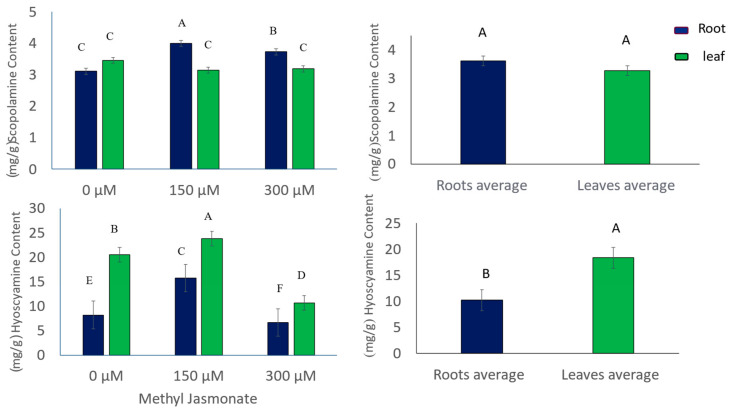
Effects of different levels of MJ on the contents of scopolamine and hyoscyamine in leaves (green) and roots (blue) on the left side and a comparison of scopolamine and hyoscyamine contents in leaf and root samples on the right side. Different letters indicate a significant difference, whereas the same letters indicate any significant difference (LSD, *p* < 0.05, *n* = 3).

In the leaf tissue, hyoscyamine levels increased significantly by 16% after treatment with 150 µM MJ. They decreased substantially by 48% after dose–response responses–responsive–response responses-responsive-response relationship (Figure 3). In the root tissue, hyoscyamine levels increased significantly by 92% after treatment with 150 µM MJ and decreased slightly by 18% after treatment with 300 µM MJ, but the changes were not statistically significant (Figure 3). This implies that hyoscyamine is more sensitive to MJ in the leaf than in the root and that hyoscyamine can be produced and transported faster than scopolamine. MJ affected the biosynthesis of these alkaloids in a tissue-specific and concentration-dependent manner in *D. stramonium*. The alkaloid levels might also depend on the duration of MJ treatment, which requires further investigation.

The hyoscyamine content in the leaves increased by 16% after elicitation with 150 µM MJ and decreased by 48% at 300 µM MJ compared to the control. Similarly, the hyoscyamine content in the roots increased by 92% after elicitation with 150 µM MJ. It decreased by 18% at 300 µM MJ compared to the control (Figure 3). The mean levels of scopolamine and hyoscyamine in the leaf and root tissues of *D. stramonium* plants showed that the mean scopolamine level was higher in the roots than in the leaves (3.61 and 3.26 mg/g, respectively).

On the other hand, the hyoscyamine level (Figure 3) was higher in the leaves than in the roots (18.3 and 10.2 mg/g, respectively).

### 2.2. The Effect of MJ on the Expression of Genes Involved in the Biosynthesis of Tropane Alkaloids

**Figure 4:**
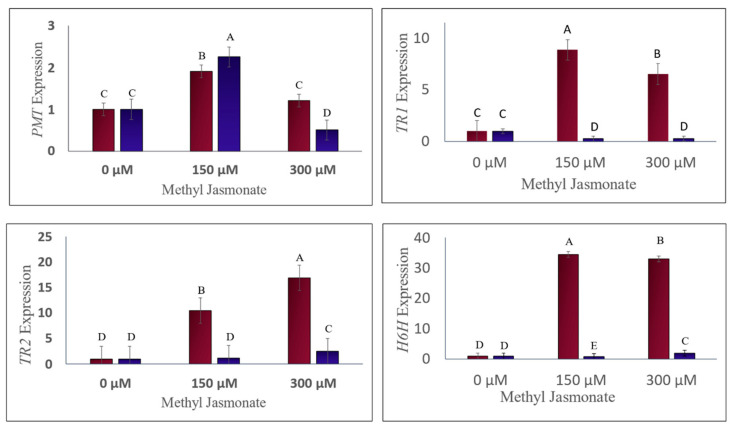
Expression Profiles of *PMT*, *TR1*, *TR2*, and *h6h* genes in *D. stramonium*. The graph illustrates the gene expression in roots (red) and leaves (blue) under normal conditions and after treatment with methyl jasmonate (MJ). Distinct letter groupings (A–E) denote statistically significant differences between the groups. Consistent letters across the groups suggest no significant difference.

The effect of MJ on the expression of four essential genes (*PMT*, *TR1*, *TR2*, and *h6h*) involved in the biosynthesis of tropane alkaloids in the *D. stramonium* plant was investigated using qRT-PCR (Figure 4). MJ concentration, tissue type (leaf or root), and their interaction significantly affected the expression of all genes except *PMT*. *PMT* catalyzes the conversion of putrescine to N-methyl putrescine, the first step in scopolamine biosynthesis (Biastoff et al., 2009) [34]. *PMT* expression was modulated by MJ concentration in a tissue-specific manner: it increased by 2.25-fold in leaves and 1.2-fold in roots at 150 µM MJ but decreased by 0.5-fold in leaves and remained unchanged in roots at 300 µM MJ compared to the control. The highest *PMT* expression was detected in the leaf sample treated with 150 µM MJ (group A), while the lowest expression was found in the leaf sample treated with 300 µM MJ (group D). *TR1* converts tropinone to tropine, an intermediate in hyoscyamine and scopolamine biosynthesis. *TR1* expression was influenced by both MJ concentration and tissue type, as well as their interaction: it decreased by 0.25-fold and 0.29-fold in leaves but increased by 8.8-fold and 6.5-fold in roots at 150 and 300 µM MJ, respectively, compared to the control.

The highest *TR1* expression was observed in the root sample treated with 150 µM MJ (group A), while the lowest expression was seen in the leaf samples treated with either 150 or 300 µM MJ (group E). *TR2* is involved in the secondary pathway of pseudo-tropine biosynthesis. *TR2* expression was also affected by MJ concentration and tissue type, as well as their interaction: it increased by 1.1-fold and 2.4-fold in leaves and by 10.4-fold and 16.8-fold in roots at 150 and 300 µM MJ, respectively, compared to the control.

The highest *TR2* expression was detected in the root sample treated with 300 µM MJ (group A), while the lowest expression was found in the leaf sample treated with 150 µM MJ and the root and leaf control samples (group D). *h6h* catalyzes the conversion of hyoscyamine and L-6-hydroxy hyoscyamine to L-scopolamine, the final step in scopolamine biosynthesis. *h6h* gene expression was also influenced by MJ concentration and tissue type, as well as their interaction: it decreased by 0.84-fold in leaves but increased by 34-fold and 32-fold in roots at 150 and 300 µM MJ, respectively, compared to the control. The highest *h6h* gene expression was detected in the root sample treated with 150 µM MJ (group A), while the lowest expression was seen in the leaf sample treated with 150 µM MJ (group E).

These results indicate that MJ treatment stimulates the expression of genes involved in the biosynthesis of tropane alkaloids in the *D. stramonium* plant, especially in roots, where the alkaloids are predominantly synthesized. *h6h*, the critical enzyme for scopolamine production, showed the most pronounced response to MJ treatment, suggesting that MJ may enhance the production of scopolamine in the *D. stramonium* plant.

The HPLC analysis showed that the alkaloid content of the leaves did not increase significantly after MJ treatment, which could be due to the short duration of the treatment before harvesting. The gene expression results revealed that *h6h*, which catalyzes the conversion of hyoscyamine to scopolamine, had the highest fold change among all genes. In contrast, *PMT*, which initiates the scopolamine biosynthetic pathway, had the lowest fold change.

The root samples treated with 150 µM MJ showed a significant upregulation in *PMT*, *TR1*, and *h6h* genes compared to the other samples, which was in agreement with the HPLC results, as these genes are involved in the biosynthesis of scopolamine and hyoscyamine (the HPLC results showed a higher concentration of tropane alkaloids at 150 µM MJ).

However, the root samples treated with 300 µM MJ showed the highest expression of *TR2*, which is involved in the secondary pathway of pseudo-tropine biosynthesis. This did not alter the conclusion, as *TR2* does not contribute to scopolamine and hyoscyamine production. This study aimed to compare the role of the aerial parts of the plant and the production of alkaloid output. The results demonstrated that the roots had a higher expression of genes related to the biosynthesis of hyoscyamine and scopolamine and were thus the primary site of production of these secondary metabolites. The expression of these genes was also detected in the aerial parts and leaves but at significantly lower levels. The expression of the genes in the leaves increased up to 2.5-fold by applying MJ treatment, while in the roots, the expression increased up to 34-fold. The MJ treatment had different effects on the expression of genes in the leaves, downregulating some and upregulating others. In contrast, in the roots, the expression of all genes increased after MJ treatment.

### 2.3. Principal Component Analysis

**Figure 5:**
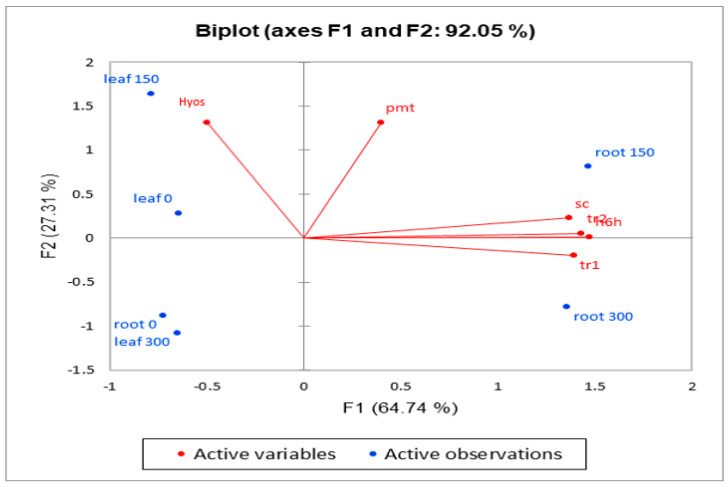
Biplot of data correlation. The x-axis represents factor 1, and the y-axis represents factor 2.

This analysis showed that the first two components accounted for 92% of the total variation. The first component (PC1) explained 64.74% of the total variation and positively correlated with the *h6h* gene. Therefore, this component was named hyoscyamine beta 6-hydroxylase expression. The second component (PC2) explained 27.31% of the variation and positively correlated with the total *PMT* content. Therefore, this component was named putrescine methyltransferase expression. Based on the results of this analysis, the data related to *PMT* and hyoscyamine were separated from the rest of the data. Considering the high values of PC1 and PC2 for our data, this method was regarded as suitable for data analysis. As shown in Figure 5, scopolamine had a high correlation with *TR1*, *TR2*, and *h6h* genes, which could indicate the effect of MJ on part of the biosynthetic pathway.

Since hyoscyamine is used in the biosynthesis of scopolamine, this could explain the negative correlation of hyoscyamine with the expression of *TR1* and *h6h* genes.

### 2.4. Correlation Test

**Figure 6:**
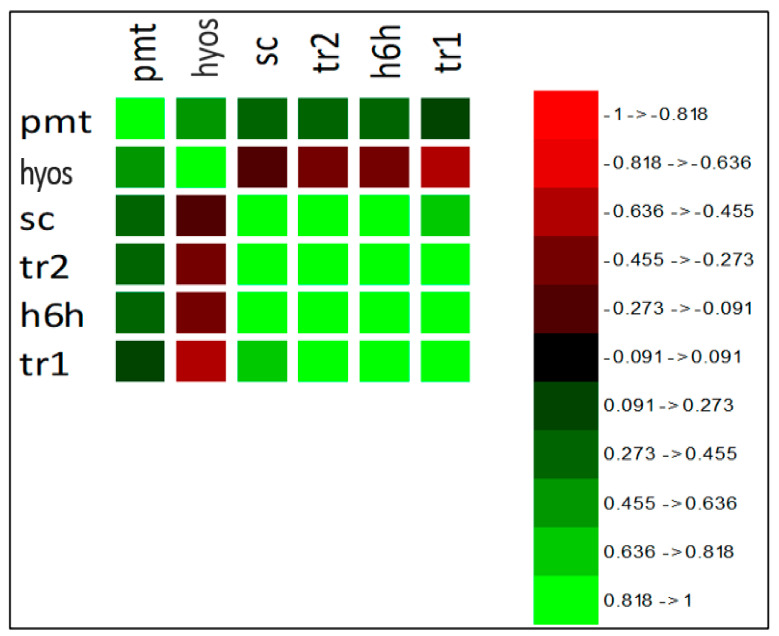
Correlation coefficients between the variables using a color scale. The darker the color, the stronger the correlation.

**Figure 7:**
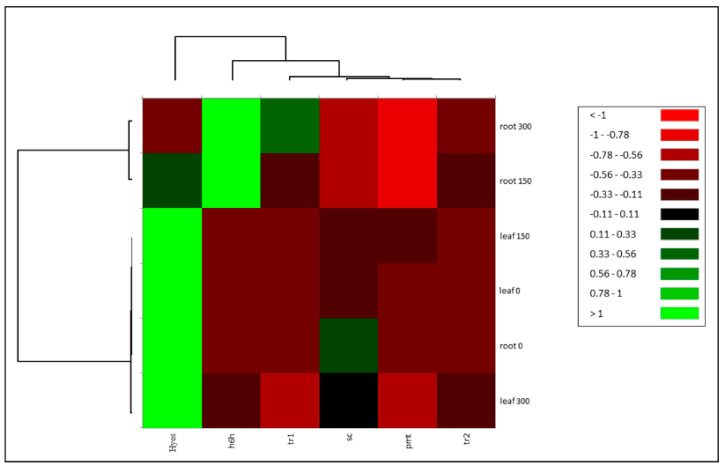
Correlation heat map of alkaloid content and conversion in *D. stramonium*. This heat map represents the correlation coefficients among various variables, illustrating the relationships within the biosynthetic pathway of *D. stramonium* alkaloids. The color’s intensity correlates with the association’s strength, whereas darker shades signify stronger correlations. Notably, the conversion of hyoscyamine to scopolamine is depicted, highlighting a significant correlation between the hyoscyamine content and the leaf tissues, as well as the root tissues, at 0 µM MJ concentration—indicative of lower scopolamine production. In contrast, root tissues treated with 300 µM and 600 µM MJ concentrations exhibit the highest scopolamine production, as reflected by their strong correlation with scopolamine levels on the heat map.

The correlation test revealed a high association of scopolamine with *TR2*, *h6h*, and *TR1* genes and a low association with putrescine N-methyl transferase (*PMT*). A heat map method (Figure 6 and Figure 7) was applied to classify the data. This suggests that hyoscyamine formed a distinct group from scopolamine, *PMT*, *TR1*, *TR2*, and *h6h*. The roots treated with 150 and 300 µM MJ also clustered separately from the other samples. Hyoscyamine, another metabolite of interest, exhibited a high correlation with *PMT* and a negative correlation with scopolamine, *TR1*, *TR2*, and *h6h*. This could be explained by the fact that *PMT* catalyzes the conversion of putrescine to N-methyl putrescine at the initial step of the biosynthetic pathway, thus influencing the synthesis of hyoscyamine. Conversely, the *TR1* gene, which mediates the transformation of tropinone to tropine in the intermediate step, is involved in synthesizing hyoscyamine but negatively correlates with it. This could be attributed to the subsequent production of scopolamine from hyoscyamine in this pathway. The drastic changes in *h6h* expression after MJ treatment resulted in the conversion of most of the hyoscyamine to scopolamine. The negative correlation of hyoscyamine with *TR1* was lower than that with *h6h*. The *h6h*, responsible for the consumption of hyoscyamine, showed a strong negative correlation.

## 3. Discussion

An investigation into MJ’s regulatory influence on the biosynthetic pathways of tropane alkaloids in *D. stramonium* was conducted. Gene expression profiling techniques demonstrated that MJ induces a tissue-specific and concentration-dependent modulation of the alkaloid profile in *D. stramonium*. These results underscore the potential of MJ as a selective elicitor in the tropane alkaloid biosynthetic pathway, providing valuable insights for optimizing these pharmacologically essential compounds.

The findings of this study are in concordance with existing literature, which documents the enhancement of scopolamine biosynthesis in response to MJ treatment across various plant taxa (Kohnen et al., 2018; Palazón et al., 2008; Wen et al., 2023) [35,36,37]. The observed increase in scopolamine production within the root tissues of *D. stramonium*, while foliar levels remained unchanged, supports the hypothesis that scopolamine synthesis is predominantly an underground process. This is consistent with research suggesting that scopolamine biosynthesis is localized to the root pericycle cells (Nakajima & Hashimoto, 1999) [38].

The mechanisms by which MJ enhances scopolamine biosynthesis in *D. stramonium* roots are not fully understood. However, it is hypothesized that MJ may modulate the expression or activity of key enzymes, such as *h6h*, which is crucial for scopolamine production. MJ could also affect the translocation or availability of the precursors necessary for scopolamine synthesis. Further studies are needed to elucidate the exact molecular mechanisms involved.

Additionally, the study highlights a dose-dependent response of hyoscyamine levels to MJ treatment, with an initial increase in foliar tissues at 150 µM followed by a decrease at 300 µM. This contrasts with the root tissue response, where hyoscyamine levels increased after 150 µM MJ treatment but showed only a marginal reduction at 300 µM, indicating a more complex interaction between MJ concentration and tropane alkaloid biosynthesis. These insights contribute to a deeper understanding of the metabolic regulation of tropane alkaloids and pave the way for future biotechnological applications in medicinal plant cultivation and alkaloid production.

The current study’s findings align with existing research, suggesting that hyoscyamine exhibits a heightened sensitivity to MJ in the leaves compared to the roots, consistent with the known biosynthetic and translocation patterns of tropane alkaloids. This observation supports the hypothesis that scopolamine and atropine are synthesized in the roots and subsequently transported to the aerial parts of the plant. The modulation of hyoscyamine content by MJ could be attributed to its influence on key enzymes, such as ornithine decarboxylase (*ODC*), *PMT*, and TRI, which are integral to the hyoscyamine biosynthetic pathway (Hedayati et al., 2020; Singh et al., 2011) [39,40]. Additionally, MJ may affect the transport and availability of the precursors and co-factors necessary for hyoscyamine synthesis, including ornithine, putrescine, and pyridoxal phosphate.

Further research is essential to elucidate the precise molecular mechanisms by which MJ affects hyoscyamine synthesis and distribution in *D. stramonium*. Future studies should focus on the transcriptional and post-translational regulation of enzymes involved in the hyoscyamine pathway and the dynamics of precursor and co-factor allocation.

This investigation contributes to the broader understanding of tropane alkaloid metabolism manipulation in plant systems. Tropane alkaloids are highly valued for their pharmacological and medicinal properties, yet their biosynthetic and regulatory pathways are complex and poorly understood. Producing these compounds in vitro and in vivo remains a significant challenge, underscoring the need for continued research in this field.

The elucidation of the tissue-specific and concentration-dependent influences of MJ on the biosynthesis and accumulation of scopolamine and atropine in *D. stramonium* sheds light on the intricate factors modulating tropane alkaloid metabolism. The identification of potential targets and mechanisms through which MJ modulates the production and transport of scopolamine and atropine in *D. stramonium* heralds new avenues for the strategic manipulation of tropane alkaloid metabolism. For instance, MJ’s enhancement of scopolamine or atropine production could be attributed to its impact on the expression or activity of pivotal enzymes within the tropane alkaloid biosynthetic pathway. Alternatively, MJ’s utility as a molecular probe could unveil the genetic and proteomic landscape governing the tropane alkaloid pathway, as evidenced by transcriptomic or proteomic alterations post-MJ treatment in *D. stramonium*.

Such methodologies could spearhead the discovery and engineering of novel genes or enzymes, propelling advancements in the production and refinement of tropane alkaloids.

In a related vein, Kang et al. (Kang et al., 2004) [41] explored the ramifications of MJ for the biosynthesis and accumulation of hyoscyamine and scopolamine in *Setaria parviflora* hairy roots. Their findings revealed that MJ escalated the levels of both alkaloids, albeit with a concomitant inhibition of root growth. Notably, MJ concentrations of 1000 and 2000 µM precipitated tissue browning within 24 h and plant mortality within 72 h, whereas lower concentrations (10 and 100 µM) were benign. The zenith of hyoscyamine and scopolamine accumulation was observed at 1000 and 2000 µM, respectively, after 24 h.

The research on *D. stramonium* has provided valuable insights into the tissue-specific and concentration-dependent effects of MJ on the biosynthesis and accumulation of scopolamine and atropine. This study confirms that MJ can significantly modulate tropane alkaloid metabolism in other Solanaceae plants. The potential of MJ in enhancing the production of scopolamine or atropine by influencing the expression or activity of key enzymes in the biosynthetic pathway is supported by similar observations in *Hyoscyamus niger*, where MJ treatments have been shown to increase the levels of these alkaloids (Albayrak et al., 2024) [42].

Furthermore, using MJ as a molecular probe to investigate the genetic and proteomic changes associated with tropane alkaloid biosynthesis is a promising approach, which could lead to the discovery of novel genes or enzymes. Studies demonstrating that MJ can induce transcriptomic and proteomic alterations corroborate this, thereby providing a deeper understanding of the regulatory mechanisms involved in alkaloid production.

The findings from Ref (Kang et al., 2004) [41] on *Setaria parviflora* hairy roots provide a comparative perspective, illustrating that higher concentrations of MJ can lead to increased alkaloid levels but can also result in adverse effects, such as tissue browning and plant mortality. This highlights the importance of optimizing MJ concentrations to balance the enhancement of alkaloid production with plant viability.

Overall, the current study contributes to the growing body of literature on the biotechnological applications of MJ in medicinal plant cultivation and offers new directions for enhancing the production of valuable tropane alkaloids through strategic metabolic manipulation.

The current study’s findings on the impact of MJ on tropane alkaloid biosynthesis in *D. stramonium* offer a nuanced perspective compared to previous research. The observed increase in atropine and decreased scopolamine in the roots after MJ treatment align with Moradi et al.’s findings [22], which suggested that 150 µM MJ was optimal for producing alkaloids in henbane hairy roots. However, the lack of significant changes in scopolamine levels in the leaves in the present study contrasts with Moradi et al.’s [22] results, where scopolamine levels increased (Dubey et al., 2021) [43]. This discrepancy may be attributed to the different species studied, as well as the duration of the experiment.

The study’s focus on the expression of *PMT* and *h6h* genes and their role in the accumulation of hyoscyamine and scopolamine is particularly insightful. The upregulation of both genes and the consequent enhancement of alkaloid levels, especially the pronounced effect on *h6h* and scopolamine, suggest that *h6h* may serve as a critical rate-limiting enzyme in the biosynthetic pathway of tropane alkaloids in *Atropa belladonna*. This agrees with some prior studies but contrasts with others, highlighting the complexity of secondary metabolite biosynthesis and the need for further research to understand the underlying mechanisms.

The study contributes to the broader understanding of MJ’s influence on the expression of biosynthetic genes and the production of tropane alkaloids. While *PMT* overexpression can increase hyoscyamine and scopolamine production in some species, this is not universally observed across all Solanaceous plants. Additionally, the role of MJ in elevating *PMT* expression and the levels of tropine and tropinone, the precursors to hyoscyamine and scopolamine, has been reported, further emphasizing the elicitor’s role in tropane alkaloid biosynthesis. However, the lack of enhancement in hyoscyamine and scopolamine levels despite *PMT* overexpression in some cases indicates that other factors, such as plant species, gene sources, vector systems, expression levels, elicitor concentrations, treatment durations, and environmental conditions, play a significant role in the biosynthetic outcomes. In conclusion, the intricate interplay between MJ concentration and gene expression underscores the complexity of secondary metabolite biosynthesis in Solanaceous plants. Future studies should aim to delineate the precise mechanisms by which MJ influences these pathways, potentially leveraging advanced genomic and biotechnological approaches to enhance the production of these pharmacologically significant compounds. The current investigation extends the understanding of MJ’s influence on the expression of critical biosynthetic genes, *PMT* and *h6h*, and the consequent levels of the tropane alkaloids hyoscyamine and scopolamine in *D. stramonium*, providing a foundation for future research and biotechnological applications.

The modulation of tropane alkaloid biosynthesis by MJ in *D. stramonium* is a complex process influenced by various factors, including gene expression, enzyme activity, and tissue-specific responses. The present study’s findings on MJ’s influence on the expression of *PMT*, *TR1*, *TR2*, and *h6h* genes are consistent with the known effects of MJ as an elicitor, which can enhance secondary metabolite production in plants.

The observed enhancement in *PMT* expression in leaf tissues at an MJ concentration of 150 µM aligns with previous research indicating that MJ can selectively induce gene expression in specific plant tissues. This suggests that MJ plays a multifaceted role in the biosynthesis of scopolamine, affecting both the early and late stages of the pathway. The responsiveness of *TR1*, *TR2*, and *h6h* to MJ treatment, particularly in root tissues, supports the notion that MJ’s regulatory function is tissue-specific and concentration-dependent.

Interestingly, the pronounced response of *h6h* to MJ treatment contrasts with earlier reports of MJ’s inhibitory effect on this enzyme’s expression and activity within the same species. This discrepancy highlights the need for further investigation into the factors modulating MJ’s impact on tropane alkaloid biosynthesis, such as variations in MJ concentration, exposure duration, and plant genotype.

The current study contributes to understanding MJ’s regulatory role in tropane alkaloid biosynthesis and suggests potential strategies for enhancing scopolamine production in *D. stramonium*. These findings have significant implications for developing optimized cultivation and genetic engineering approaches to increase the yield of scopolamine, a tropane alkaloid with critical therapeutic applications.

The role of *PMT* in the biosynthesis of tropane alkaloids, particularly scopolamine, is a critical area of research within the *Datura* genus and other Solanaceae species. *PMT* catalyzes the conversion of putrescine to N-methyl putrescine, initiating the tropane alkaloid biosynthetic pathway. This step is crucial, as it directs the flow of metabolites toward producing tropane alkaloids, such as scopolamine and hyoscyamine.

The isolation and characterization of *PMT* cDNAs from various species, including Nicotiana tabacum, Hyoscyamus niger, and Atropa belladonna, have shed light on the enzymatic processes which lead to alkaloid production. For instance, the overexpression of *PMT* in *D. stramonium* has been shown to enhance scopolamine production, which aligns with the findings from Ref (Moyano et al., 2003) [44]. However, the same overexpression strategy did not significantly increase tropane alkaloid accumulation in *Atropa belladonna*, suggesting that species-specific regulatory mechanisms are at play in alkaloid biosynthesis.

A notable study by Zhang et al. (2004) (Zhang et al., 2004) [45] demonstrated that the co-expression of *PMT* with *h6h*, another critical enzyme in scopolamine biosynthesis, led to a substantial increase in scopolamine accumulation in the hairy roots of *Hyoscyamus niger*. This finding highlights the potential of gene co-expression strategies to optimize the biosynthetic yield of pharmacologically important alkaloids.

In line with these studies, transferring the *PMT* gene from Nicotiana tabacum to Datura metel using a binary vector resulted in increased levels of hyoscyamine and scopolamine in the transformed roots of *Datura metel*. In contrast, the same genetic modification in *Hyoscyamus muticus* primarily elevated hyoscyamine levels. Our current investigation supports these observations, indicating a strong correlation between *PMT* gene expression and hyoscyamine content, while the association with scopolamine appears weaker.

These differential effects underscore the complexity of the tropane alkaloid biosynthetic pathway and suggest that factors beyond gene expression, such as enzymatic efficiency and metabolite flux, may influence the accumulation of these compounds within various species of the Solanaceae family. The collective research efforts present a strong case for the strategic manipulation of key enzymatic genes to enhance the production of tropane alkaloids, which could significantly advance our understanding of plant secondary metabolism and improve the biotechnological production of these valuable compounds for pharmaceutical use.

The modulation of tropane alkaloid biosynthesis by MJ in *D. stramonium* involves various genes and their expression levels. Our study’s findings on the effects of MJ on *PMT*, *TR1*, *TR2*, and *h6h* gene expression provide valuable insights into the complex regulatory mechanisms of tropane alkaloid synthesis.

The increase in *PMT* expression at 100 µM MJ concentration and the subsequent augmentation in tropine and tropinone levels observed in our study are consistent with Deng’s findings (Deng, 2005) [46]. However, the contrasting responses of hyoscyamine and scopolamine levels following 150 µM treatment highlight the intricate nature of MJ’s modulatory effects, which may not always correlate directly with substrate availability and enzyme expression levels.

The lack of a significant increase in hyoscyamine and scopolamine levels despite the quintupled methyl putrescine levels reported by Zhang et al. suggests that the mere availability of substrates does not guarantee enhanced production of the end alkaloids. This aligns with our observations, indicating that the role of *PMT* in the biosynthetic pathway is more nuanced than merely providing the substrates for downstream enzymes.

Moradi et al.‘s [22] exploration of MJ’s impact on *h6h, PMT1,* and *PMT2* expression in chamomile plants also provides a parallel to our findings, where *h6h* expression remained unchanged in the leaves, and *PMT* expression was detected in both treated and untreated leaves. The differences in gene expression and alkaloid accumulation between the studies could be due to the different treatment durations and the inherent interspecies variability in response to MJ.

## 4. Materials and Methods

### 4.1. Plant Material

Seeds of *Datura stramonium* L., obtained from the Research Institute of Forests and Rangelands, Tehran, Iran, were sown in pots filled with a 1:1 volume ratio mixture of soil and vermiculite. The seedlings were consistently irrigated and cultivated under controlled greenhouse conditions, which included a 12 h photoperiod, a stable temperature of 25 °C (±2 °C), and a light intensity of approximately 120 µmol·m^−2^·s^−1^ using cold fluorescent light 60 cm above the plots. There was one fluorescent lamp for every three pots. The pots used had a radius of 11 cm, ensuring adequate space for initial growth. The seedlings utilized in this study were carefully selected at a uniform developmental stage of 6 months. This age represents a critical phase in the plant’s growth, where the biosynthesis of tropane alkaloids is active, providing an optimal window for assessing the genetic and metabolic responses under our experimental conditions.

### 4.2. Methyl Jasmonate Induction

The study examined the effects of MJ on the biosynthetic pathways of scopolamine and hyoscyamine in *D. stramonium*. MJ treatments were administered at 150 µM and 300 µM concentrations through foliar spray at the 50% flowering stage. The experimental framework included three biological replicates for each treatment group, with each replicate consisting of three plants, totaling nine plants per MJ concentration. This design was implemented to reinforce the findings’ statistical robustness and comprehensively evaluate MJ’s influence.

This investigation utilized taproots from *D. stramonium*, acknowledging their crucial role in the biosynthesis and storage of tropane alkaloids. Mature leaves were also selected due to their elevated metabolic activity in alkaloid production compared to young leaves, ensuring an accurate representation of the plant’s alkaloid profile after MJ treatment. Following a 24-h MJ exposure, leaf, and root tissues were promptly harvested, cryopreserved in liquid nitrogen, and stored at −80 °C for subsequent analysis. This protocol was meticulously followed to maintain the integrity of the samples for biochemical assessment.

### 4.3. RNA Isolation and Reverse Transcription

According to the manufacturer’s instructions, total RNA was isolated from 0.1 g of plant tissue using RNXTM-PLUS solution (GeneAll Biotechnology, Seoul, Republic of Korea). The RNA samples were treated with *DNase I* (Fermentas, GeneAll Biotechnology, Seoul, Republic of Korea) to remove any genomic DNA contamination. Nanodrop spectrophotometry (Thermo Fisher Scientific, Waltham, MA, USA) and agarose gel electrophoresis verified the RNA quality and quantity [47]. The RNA samples were expected to have an A260/A280 ratio of 1.8–2.0 and an A260/A230 ratio of 2.0–2.2 and to show clear 18S and 28S ribosomal RNA bands on the gel. cDNA was synthesized from 1 µg of RNA using an Easy cDNA synthesis Kit (Applied Biological Materials, Thermo Fisher Scientific, Waltham, MA, USA), following the manufacturer’s protocol.

### 4.4. Primer Design and Quantitative Real-Time PCR (qRT-PCR) 

The primer design for *PMT*, *TR1*, *TR2*, and *h6h* genes, which are integral to the alkaloid biosynthetic pathway, was accomplished using Oligo Primer Analysis Software v. 7 (https://www.oligo.net/). This version of the software is equipped with advanced algorithms for optimal primer selection, ensuring specificity and efficiency in PCR amplifications. (Figure 1). The Oligo Calculator and Oligo Analyzer software (https://www.idtdna.com/pages/tools/oligoanalyzer accessed on 7 May 2024) analyzed the primer sequences for hairpin, hetero-dimer, and self-dimer formation. The Primer-BLAST software (https://www.ncbi.nlm.nih.gov/tools/primer-blast/ accessed on 7 May 2024) was used to check the specificity of the primers. The forward and reverse primer sequences used for qRT-PCR are shown in Table 2.

qRT-PCR was performed in a Rotor-Gene 6000 series instrument (QIAGEN, Hilden, Germany) with SYBR Green Master Mix 2X, following the manufacturer’s instructions. Each cycle consisted of three steps: 95 °C for 15 s, 59–65 °C (based on the annealing temperature from PCR gradient results) for 20 s, and 72 °C for 20 s. The reactions were repeated with three technical replicates for statistical analysis. The 2^−ΔΔCT^ method was used to calculate the relative expression of the different genes (Schmittgen & Livak, 2008) [48].

### 4.5. Preparation of Extracts for HPLC Analysis

The alkaloids of interest were isolated from 2.0 g of a wet sample using a chloroform-based extraction method, as described by Djilani and Legseir (2005) and Hosseini et al. (2011) (Djilani & Legseir, 2005; Hosseini et al., 2011) [49,50]. The sample was sonicated for 15 min with 10 mL of extraction solution (1:5:1 of chloroform, methanol, and 25% NH_4_OH) and filtered twice with a paper filter. The filtrates were combined, dried with nitrogen gas, and acidified with 3 mL chloroform and 2 mL H_2_SO_4_. After shaking for 1 min, the residue was dried again. The aqueous phase was adjusted to pH 11 with NH_4_OH and filtered with 1 mL of chloroform and Na_2_SO_4_. The filtrate was dried, reconstituted with 0.5 mL of methanol, and stored at −8 °C until HPLC analysis. HPLC analysis was performed on 12 samples, with two replicates for each treatment. The scopolamine, atropine, and hyoscyamine peaks, which are racemic isomers, were eluted at 3 and 3.7 min, respectively.

### 4.6. High-Performance Liquid Chromatography (HPLC)

Scopolamine and hyoscyamine standards were obtained from the Medicinal Plants Research Institute of Shahid Beheshti University. Chromatographic analyses were performed on a Knauer UHPLC system (Berlin, Germany) model PLATIN blue with a photodiode array detector (PDA). A NUCLEODUR 100-5 C18 ec column (5 μm, 250 mm, 4.6 mm) was used for the separation in isocratic mode with a mobile phase of sodium phosphate buffer and acetonitrile (80:20, *v*/*v*), pH 3.0. The flow rate and the injection volume were 1 mL/min and 20 μL, respectively. The column temperature was ambient, and the detection wavelength was 210 nm. Scopolamine and hyoscyamine were eluted at about 3 and 3.7 min, respectively.

### 4.7. Principal Component Analysis

Principal component analysis (PCA) was applied to reduce the data volume and extract the primary sources of variation in the samples (Abdi & Williams, 2010) [51]. The PCA also revealed the correlation between real-time and HPLC measurements for the untreated and treated samples. Using Heatmap Ly, a heat map was constructed to visualize each variable’s mean and standard deviation for different treatments.

### 4.8. Statistical Analyses

The analytical phase involved using IBM SPSS Statistics as the principal analytical tool for comprehensively evaluating and interpreting the dataset. Statistical techniques and methodologies within SPSS were applied to perform a holistic analysis. Detailed examination of the SPSS-generated outputs facilitated an in-depth exploration of the dataset, identifying the underlying patterns, trends, and correlations and deriving substantive insights. Variances within the data were scrutinized to evaluate their statistical significance, discerning whether they represented meaningful differences or were attributable to random fluctuations. This rigorous analytical strategy ensured that the conclusions were anchored in solid statistical evidence, supporting enlightened decision-making based on empirical findings.

## 5. Conclusions

This study elucidated the modulatory role of MJ, a phytohormone, in the biosynthesis and distribution of tropane alkaloids, specifically scopolamine and hyoscyamine, in *D. stramonium*. Our findings revealed that MJ influences these alkaloids in a tissue-specific and concentration-dependent manner. Notably, at a concentration of 150 µM, MJ significantly enhances the production and accumulation of both alkaloids in the leaves and roots. Conversely, a higher concentration of 300 µM proved to be counterproductive. The upregulation of the *h6h* and *PMT* genes, pivotal in the tropane alkaloid biosynthetic pathway, was predominantly observed in the roots, indicating a strategic augmentation of alkaloid biosynthesis in this organ. The optimal concentration of 150 µM MJ was found to boost the expression of *h6h*, *TR1*, and *PMT* genes, with the most substantial effect on *h6h*, which experienced a 34-fold increase in expression. This contrasts with the more modest 2.7-fold increase observed for *PMT*. Our correlation analysis indicates a strong positive relationship between scopolamine levels and the expression of *TR1* and *h6h* genes, situated in the central and terminal stages of the biosynthetic pathway, respectively. In contrast, hyoscyamine levels negatively correlate with these genes, as their upregulation promotes the conversion of hyoscyamine into scopolamine. These insights highlight MJ’s potential in fine-tuning tropane alkaloid metabolism in *D. stramonium*, optimizing the yield of these pharmacologically essential compounds. Moreover, the study sheds light on the intricate biosynthetic and transport mechanisms governing scopolamine and hyoscyamine production, setting the stage for innovative genetic and metabolic engineering strategies to enhance tropane alkaloid yields. While the precise mechanisms of MJ’s influence on tropane alkaloid levels in *D. stramonium* remain to be fully deciphered, this research provides a foundational understanding for future molecular and biochemical investigations into plant tropane alkaloid metabolism, opening new pathways for the enhanced production of these valuable alkaloids.

## Figures and Tables

**Table 1:** Mean concentrations of scopolamine and hyoscyamine in *D. stramonium* tissues.

MJ Concentration (µM)	Scopolamine (Leaf)	Scopolamine (Root)	Hyoscyamine (Leaf)	Hyoscyamine (Root)
0	3.05 ± 0.09	3.11 ± 0.15	20.54 ± 0.79	8.19 ± 0.22
150	3.15 ± 0.06	4 ± 0.06	23.85 ± 0.85	15.75 ± 0.06
300	3.19 ± 0.15	3.73 ± 0.12	10.68 ± 0.1	6.66 ± 0.22

Note: Values represent the mean concentrations of scopolamine and hyoscyamine measured in milligrams per gram (mg/g) of leaf and root tissues from *D. stramonium* plants 24 h post-methyl-jasmonate (MJ) treatment. The standard error (SE) is indicated for each sample.

**Table 2:** Specific primers used in qRT-PCR.

Target Gene	Primer Sequences (Sequence in 5′-3′ Direction)
*h6h*	F: 5′GAACGACGCTGTAATGAGGAG3′ R: 5′GTCAACTTCCTCACTTCCACT3′
*PMT*	F: 5′GCTTCGTTATCCTACCGTTG3′ R: 5′ACGAGGATCATTAAAGTTAGCC3′
*TR1*	F: 5′CCTTGTTACTGGTGGCTCTAA3′ R: 5′CCAAATTTCAAGGCATTCGT3′
*TR2*	F: 5′AGGAGCAATGGATCAACTCA3′ R: 5′TCGACCAGAGAAGTTGCAATA3′
*TUB*	F: 5’CCATAAGTTTGATCTCATGTATGC3 R: 5’CAAGGTCCTCACGAGCCT3′

## Data Availability

The data presented in this study are available on request from the corresponding author.
